# Beyond Conceptual Knowledge: The Impact of Children’s Theory-of-Mind on Dyadic Spatial Tasks

**DOI:** 10.3389/fpsyg.2016.01635

**Published:** 2016-10-20

**Authors:** Karine M. P. Viana, Imac M. Zambrana, Evalill B. Karevold, Francisco Pons

**Affiliations:** ^1^Department of Psychology, University of OsloOslo, Norway; ^2^Department of Special Needs Education, University of OsloOslo, Norway; ^3^The Norwegian Center for Child Behavioral DevelopmentOslo, Norway

**Keywords:** theory-of-mind, spatial task, cognitive performance, dyadic interaction, children

## Abstract

Recent studies show that Theory of Mind (ToM) has implications for children’s social competences and psychological well-being. Nevertheless, although it is well documented that children overall take advantage when they have to resolve cognitive problems together with a partner, whether individual difference in ToM is one of the mechanisms that could explain cognitive performances produced in social interaction has received little attention. This study examines to what extent ToM explains children’s spatial performances in a dyadic situation. The sample includes 66 boys and girls between the ages of 5–9 years, who were tested for their ToM and for their competence to resolve a Spatial task involving mental rotation and spatial perspective taking, first individually and then in a dyadic condition. Results showed, in accordance with previous research, that children performed better on the Spatial task when they resolved it with a partner. Specifically, children’s ToM was a better predictor of their spatial performances in the dyadic condition than their age, gender, and spatial performances in the individual setting. The findings are discussed in terms of the relation between having a conceptual understanding of the mind and the practical implications of this knowledge for cognitive performances in social interaction regarding mental rotation and spatial perspective taking.

## Introduction

The development of ‘theory of mind’ (ToM), which is the ability to understand the nature, origins, and consequences of the mind (beliefs, intentions, desires, feelings, etc.) in the self and others, has been investigated extensively (e.g., Wellman et al., 2001; Shahaeian et al., 2011; Harris et al., 2016). However, much less is known about the implications of ToM for children’s social and cognitive development (e.g., Harris, 2006; Grüneisen et al., 2015). On one hand, recent studies have shown that children’s ToM is positively associated with their overall prosocial behaviors and social competences (Caputi et al., 2012; Roazzi et al., 2013; Farina and Belacchi, 2014). On the other hand, the implications of ToM for children’s cognition has received less attention and the findings are typically inconsistent (Meins et al., 2006; Veneziano et al., 2008; Guajardo and Cartwright, 2016). Furthermore, albeit it is well documented that on a range of cognitive problems children obtain better performances when solving these together with a partner (e.g., Doise and Mugny, 1984; Tversky and Hard, 2009), to the best of our knowledge, no study has addressed the degree to which individual differences in ToM can account for cognitive problem resolving performances in dyadic settings. In light of the particular reliance on collaborative task performances in the awaiting and modern academic and vocational life, understanding whether collaborative tasks depend on individual abilities can have both practical and pedagogical implications. The present study therefore aims to investigate whether ToM can explain children’s performance in a dyadic spatial transformation task which demands the cognitive ability to mentally rotate objects and the coordination of different viewpoints.

When the term ‘theory-of-mind’ was originally introduced, it was thought of as the competence to attribute mental states to self and others, involving the ability to theorize about others’ mind by making inferences regarding mental phenomena (Premarck and Woodruff, 1978). It was thus recognized as a socio-cognitive skill enabling human beings to predict, explain and manipulate others’ actions and representations. Traditionally, false-belief tasks, based on the attribution of a mistaken belief, have been central in assessing children’s ToM capacities (Wimmer and Perner, 1983). Today, however, it has become more and more common to consider ToM through a wider lens; not only involving the understanding of belief and knowledge, but also encompassing the competence to conceptually understand intentions, desires, and emotions (e.g., Astington, 2001; Wellman et al., 2001; Pons et al., 2004; Dunn, 2006; Shahaeian et al., 2011).

The first studies in the ToM field presented strong evidence for the progress children obtain between 3 and 5 years of age on classical false-belief, appearance-reality, and Level-2 visual perspective taking tasks (Flavell et al., 1983; Flavell, 2004). Albeit important milestones in ToM development occur in the preschool years, the knowledge about mental states continues to increase later on (Flavell, 2004). Research has shown that from infancy to adolescence, ToM develops from a “peripheral and superficial” understanding of rather visible or non-reflective dimensions of the mind (e.g., recognition of basic emotions, understanding of first order false-beliefs and impact of desires on emotions) to a more “central and deeper” understanding of more invisible or reflective dimensions of the mind (e.g., understanding of moral and mixed emotions, of second order false-beliefs and double-bluffs; Pons et al., 2009).

Different directions of research emerged from these early works on trends in ToM development. These studies have been exploring, for instance, antecedents that might contribute to ToM development, intra and intercultural differences, and real world consequences of ToM abilities (e.g., Flavell, 2004; Shahaeian et al., 2011). It is well documented that ToM development depends on many social and cognitive factors, such as language, intelligence, executive function, attachment, and relationships with peers (e.g., Cutting and Dunn, 2006; Pons et al., 2014). Recent studies have also found positive impacts of ToM on social competences at the ages of 3–6 years and psychological well-being at the ages of 8–12 years (e.g., Farina and Belacchi, 2014; Bender et al., 2015). However, the implications of understanding mental states for children’s cognition remain unclear. For instance, Veneziano et al. (2008) found that 6–7 year-olds with higher ToM test scores were better able to express epistemic states when they narrated a story. A longitudinal study conducted by Guajardo and Cartwright (2016) tested children at 3–5 years and later at 6–9 years and showed that those who had better understanding of other’s perspectives were more aware of their thoughts involved in reading. Lecce et al. (2010) found the same results in a study assessing children between 9 and 10 years of age. On the other hand, Meins et al. (2006) argue that between 6 and 9 years of age, having ToM capacities, measured through conceptual tasks, is different from being able to use it either to narrate a book or to describe friends. Likewise, Guajardo and Cartwright (2016) showed that false-belief understanding did not contribute uniquely to reading comprehension. Together, this suggests at least two gaps when it comes to understand the role of ToM for children’s cognition. First, previous studies do not cover a broad measure of ToM that also includes the understanding of desires and emotions; and second, there is still a need to explore other cognitive dimensions potentially influenced by ToM in school-aged children that go beyond the use of mental terms and reading comprehension. One such dimension is the performance on cognitive tasks completed together with peers.

Studies on the impact of ToM on cognitive performances in dyadic interaction are rare, and have especially focused on false-belief reasoning and the process (rather than the outcome) of cooperation. If on one hand it has been shown that ToM works as a powerful social tool that facilitates children’s interactions with peers (Moore and Frye, 1991), it remains unclear whether ToM has implications for the cognitive outcome produced in social interaction. For instance, Grüneisen et al. (2015) recently found that 6-year-olds could use first and second order false-beliefs to coordinate actions with peers, showing that recursive mind-reading is an important component of dyadic interaction. Similarly, Flobbe et al. (2008) demonstrated that 8–10 year-olds passing a second order false-belief task are able to apply this when playing a strategic game with a peer. Curry and Chesters (2012) showed that adults scoring lower on a self-report measure of autistic traits and understanding of other’s minds were also less successful at coordinating their behaviors with others in coordination games. These researchers subsequently called for studies using a broader range of ToM measures to investigate the impact of children’s understanding of the mind on their performances in dyadic settings. Investigating how children solve a spatial transformation task in a dyadic situation might be particularly relevant in this context because it requires both the cognitive ability to mentally rotate objects and the adoption of the spatial perspective of someone else (Kessler and Thomson, 2010).

Spatial abilities comprise activities such as perception of horizontality, mental rotation of objects, or location of simple figures within complex figures (Linn and Petersen, 1985). Specifically, spatial transformation demands the ability to mentally rotating objects and making transformations in their positions based on a specific referential mark (Hegarty and Waller, 2004). Piaget and Inhelder (1952) focused in particular on one aspect of spatial relations called “coordination of perspectives,” which refers to the ability to identify the appearance of an object as something dependent on the spatial position from which they are viewed. Based on the classical “three mountains task,” they found that children younger than 6 years locate objects with respect to their own points of view, and it is only between 7 and 9 years of age, when children reach the concrete operational stage, that they would be aware of other perspectives than their own and thus deal with an external frame of reference (Piaget and Inhelder, 1952). Spatial relations, therefore, comprises both the cognitive process of projecting relationships between objects, and the social process of understanding the relation between two different perceptions, as exemplified by the “If I were in your place I would see what you see” line of thinking (Fishbein et al., 1972). Flavell et al. (1981) claimed that even under the age of 3, children recognize that people can perceive different objects at the same time (Level-1 perspective taking) but they have difficulties with recognizing that they can see the same object from different perspectives (Level-2 perspective taking). This more sophisticated ability is likely to be developed around 5 years of age. Newcombe and Huttenlocher (1992), for instance, tested children between 3 and 9 years of age and found that children as young as 5 years can take the spatial perspective of others when the task does not entail conflict between two frames of reference.

The Piagetian paradigm presented strong evidence for the role of socio-cognitive conflicts on the development of coordination of perspectives. In the “three mountains task,” children have to visualize themselves in a different position and these conflicting representations within the individual promote a breakdown in the cognitive equilibrium that boosts a reinterpretation of the object (Zapiti and Psaltis, 2012). However, in the “three mountains task,” the perceptions were not confronted by someone else. Doise and Mugny (1984) contributed enormously to this issue by considering the spatial coordination not only as an intra-individual process but also as an inter-individual one. Based on a critical review of Piaget and Inhelder’s (1952) work, they proposed a series of experiments where the coordination of real viewpoints could take place. They tested children between 5 and 8 years of age in a spatial transformation problem called “The reconstruction of the village task,” involving both an individual and a dyadic condition. The findings demonstrated a positive impact of peer collaboration on spatial performances as children progressed on the task after they have worked with a partner. The authors argue that when solving a spatial task individually, children have to create intra-individual cognitive conflicts to envision and derive at different solutions, and that this could be less powerful than collaborative settings where the inter-individual conflict and the mutual action context promote subsequent individual progress. Moreover, it could be more effective if each member of the dyad has access to only one part of the resources needed to complete the task (Buchs and Butera, 2004). In a recent study, Zapiti and Psaltis (2012) applied the same “village task” used by Doise and Mugny and tested children between 6.5 to 7.5 years of age to analyze the impact of interaction types on task performance. They found that the pair composition in terms of the children’s gender and spatial knowledge affected the expression of point of view and the type of the socio-cognitive conflict that emerged. In a meta-analysis on gender differences in spatial ability, Linn and Petersen (1985) demonstrated how gender relates to spatial performance by showing that males are better than females in mental rotation problems and that the magnitude of this difference is smaller in spatial visualization. The authors also pointed out that the impact of gender might vary depending not simply on the task type but also on the age range of the participants (e.g., Voyer et al., 1995; Yilmaz, 2009).

Thus, even though previous research has demonstrated a positive impact of peer collaboration on spatial performances both with children and adults (Doise and Mugny, 1984; Tversky and Hard, 2009), more studies are needed in order to deepen our understanding of the mechanisms underlying spatial performance in dyadic settings. Because the “village task” demands the cognitive ability to mentally rotate objects and the coordination of different viewpoints, they can be particularly fruitful for the purpose of examining whether broader ToM capacities play a role in children’s spatial performance in social interaction. Therefore, the current study addresses two main questions: (1) whether children improve their performance when resolving a spatial transformation task with a partner as compared to alone; (2) and to what extent children’s achievements on ToM tasks explain their spatial performances in a dyadic setting. The reasons for focusing on a dyadic setting are twofold: the need to understand potential mechanisms related to individual differences in dealing with spatial problems in social interaction; and the intention to explore the impact of ToM on an advanced cognitive problem, as the performance in the dyadic condition implies not only mental rotation of objects but also the coordination of different hands on spatial perspectives.

One could argue that the village task is a perspective taking problem in itself, so why investigate whether ToM impacts another perspective taking task? First, in this study ToM is not measured based solely on perspective taking ability but as a broad competence including the understanding of beliefs, desires, and emotions (e.g., Shahaeian et al., 2011). Moreover, the “village task” cannot be reduced to its perspective taking dimension. Different from the “three mountains” (Piaget and Inhelder, 1952) and other classical perspective taking tasks, such as the picture and turtle tasks (Masangkay et al., 1974), in the dyadic version of the “the village task” a child can be confronted by the other, so that both children have to deal with two socio-cognitive operations at the same time: (1) the mental rotation of the objects based on an external frame of reference; (2) the perspective of the other child about the position of the objects in relation to the referential mark. When confronted with another spatial representation, the child is challenged to make some changes in his own spatial representation, and as Gopnik and Astington (1988) suggested, it is much easier to ignore your own contradictions than ignore the contradictions between your own representation and the representation of others. Previous studies have shown that adopting others’ perspectives remains cognitively demanding even for adults, especially when the perspectives are conflicting (Keysar et al., 2000; Epley et al., 2004; Qureshi et al., 2010). Surtees et al. (2011) tested adults and children between 6 and 11 years of age and found that those who succeed on direct tasks of Level-2 perspective taking showed no evidence of this competence when it was measured in an indirect task where the participants where not explicitly asked about what the partner was seeing. This is also the case with the “village task” in which the participants are encouraged to work together but there is no explicit question about the perspective of the other, though the children need to coordinate their spatial representations to find the solution to the problem. Consequently, we are not applying two simple perspective taking tasks. In addition, the aim is not to assess whether ToM and spatial performance are related competences, but to examine specifically to what extent the performances on classical ToM tasks with different levels of complexity and where the child attributes mental or emotional states to a character in a fictional scenario (without being confronted with another’s perspective) can explain the variation in spatial performances in an interactional scenario where the spatial representation of one child can be confronted by that of the other child. In other words, does a broad conceptual knowledge about the mind have implication for children’s cognition in the domain of a dyadic spatial task?

In accordance with previous studies, the first hypothesis is that children perform better on the Spatial task in the dyadic setting compared to when doing it by themselves, even when we consider age and gender. Because resolving the Spatial task together with a partner depends on mental rotation of objects and understanding of the other’s point of view, the second hypothesis is that children’s ToM has a positive impact on spatial performances in the dyadic version of the task, even after taking into account age, gender, and the performance in the individual condition. We expect the results to contribute to the fields of ToM development and social development in at least three ways: by consolidating previous results showing that children take advantage from dyadic setting when resolving a cognitive problem; by originally informing on the role of individual differences in ToM on children’s spatial performances in a dyadic setting (illuminating potential mechanisms underpinning spatial abilities in social interactions); and by pointing out a link between conceptual understanding of the mind and its practical implications on children’s cognitive performance in the domain of spatial transformation abilities.

## Materials and Methods

### Participants

Initially, 120 parents were contacted through two middle-class private schools in Recife (Brazil). The parents of 90 children (75% of the invited) signed a consent form that informed on the study aims and procedures, allowing their children to be asked to participate. Subsequently, all children invited agreed to participate in the study. The Norwegian Social Science Data Service and the Ethic Committee in Brazil approved the project.

To avoid floor and ceiling effects, children who did not succeed on the simplest item in the individual condition of the Spatial task (*n* = 14), as well as those who achieved the maximum score in the individual setting (*n* = 10) were excluded from the sample (Doise and Mugny, 1984). This ensured that the children could have the same minimum level and that they could also progress on the task. There were equal number of boys and girls among those who failed on the first item and 12 children in the youngest group. Amongst the children who achieved the maximum score, four were girls, six were boys, and all of them were in the oldest group. This is consistent with previous findings (e.g., Doise and Mugny, 1984), as younger children failed more often than the older ones and only children in the oldest group achieved the maximum score. Thus, the final sample included 66 typically developing children (32 boys; 34 girls) between 5 years 7 months and 9 years 8 months (*M* = 89.94 months; *SD* = 13.09 months) with Portuguese as their native language. In order to obtain more variation in terms of ToM competence (the younger group with ToM in progress and the other with well-established ToM), children were divided into two groups according to their age (*n* = 36 in the Younger group: 5;7–7;5 years; *n* = 30 in the Older group: 7;6–9;8 years). Because we wanted to facilitate that children would work together – and because asymmetry in terms of knowledge and gender might create competitive relationship instead of collaboration (Buchs et al., 2004; Sommet et al., 2015) – the dyads consisted of children of the same gender, similar age, from the same classroom, and with similar performances on the individual version of the Spatial task (*SD* = 0.84) and the ToM tasks (*SD* = 2.19). For the same reason, we wanted to ensure that the children in the dyads were neither best friends nor not friends, so that information from the children’s ranking of their friends in the classroom was also used when composing the dyads.

### Procedure, Tasks, and Scoring

The data collection consisted of three sessions carried out at the children’s schools. In the first session, the children were tested individually on the Spatial task. In the second session, the children completed the ToM tasks, and in the third and last session, they participated in the dyadic version of the spatial problem. Each session lasted around 10 min, with an average interval of 15 days between each session.

#### Spatial Task

Children were first tested individually in an adapted version of the spatial transformation task “the reconstruction of the village,” developed by Doise and Mugny (1984) and derived from Piaget’s famous “three mountains” task (Piaget and Inhelder, 1952). The task material included a miniature village placed on a model cardboard (50 cm by 50 cm), which was fixed on a table, and comprised a lake (the referential mark) and three or four houses (i.e., based on task complexity, which is described below) with different colors and marked with doors on one side. On a different table, offset 90° from their left, children could see another cardboard also marked with a lake on it. They received three or four houses equivalent to the ones previously placed by the researcher on the model cardboard, and they were instructed to make a similar village. In order to emphasize the referential mark, the experimenter said that if a man comes out of the lake, he would find the houses in the same positions as the ones in the model constructed by the experimenter. Chairs were placed in such a way that the children could not move beyond a limited area.

**FIGURE 1:**
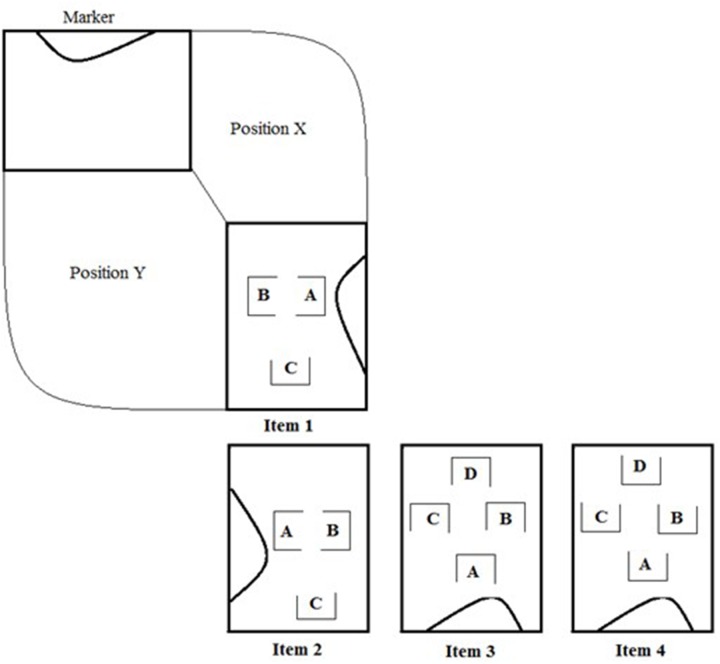
**Spatial task**.

**Figure 1** gives an overview of the task. There were four different items with increasing complexity. The simplest item had three houses with no rotation required. The second demanded a rotation of 90° and an inversion of the left-right and front-back orders of the houses. The third and fourth items had four houses and both required 180° rotations and inversions of the left-right and front-back orders. After the completion of each item, children were oriented to move to the opposite side of the cardboard to check whether or not they wanted to make changes to their villages. When solved individually, this part of the procedure generated an intra-individual cognitive conflict, as the child could look at the same village from different perspectives (Doise and Mugny, 1984).

The same four items were applied in the dyadic condition, but in this situation children were placed in different face-to-face positions (position X and position Y in **Figure 1**). This required them to coordinate their viewpoints to make a copy of a village, which entails an inter-individual cognitive conflict, as it involved looking at the same village from different angles (Doise and Mugny, 1984). To make sure that one child would not act alone, the dyadic condition operated with interdependent resources (Buchs and Butera, 2004), so that each child received only a certain number of houses (either one or two) and were only allowed to touch and move their “own” houses. To move the houses of the “other” child, the children had to convince the partner to do this, providing opportunities for negotiations within the dyad.

The same scoring method, based on the original work of Doise and Mugny (1984), was applied for both the individual and dyadic conditions. The children first got a spatial score for each item of the Spatial task. Children showing no compensation (NC) got 0 points. They did not manage to mentally rotate the cardboard and just reproduced the perceptual tableau that they were able to observe without making any inversion regarding the position of the houses. Children who displayed partial compensation (PC) received one point, meaning that they achieved one of the inversions required, either the right-left order or the front-back order, but not both. Children who demonstrated total compensation (TC) got two points, and this involved correct transformation of both dimensions (left-right and front-back) simultaneously. Subsequently, in both conditions, a total sum score was calculated from the points on the four items, therefore could vary from zero to eight in each condition of the spatial task. Because two dyads did not reach an agreement regarding the resolution of the problem, the score was computed for each child separately in both conditions. Thus, the score in the dyadic setting represents an individual result of the social interaction.

#### Theory of Mind Task

Children were tested individually for their ToM with items extracted from the Theory of Mind Test (TMT; Pons and Harris, 2002), and the Test of Emotion Comprehension (TEC; Pons and Harris, 2000). Both tests are the result of an extensive review of the developmental literature and of a selection of the most common tasks used to assess children’s ToM. Giménez-Dasí et al. (2016) have also combined these two tests to obtain a broad measure of ToM. However, the authors used a short version of the two tests by reducing the number of items and keeping all the components. In addition, they applied the tests in two separate sessions. Because we had an extensive data collection, we applied the TEC and the TMT in the same session which, in turn, required the exclusion of some components. This was a strategy to ensure that the children would be concentrated and motivated during the assessment, and reducing the number of items would still make the tests very lengthy. Moreover, more items per component should be more reliable than fewer items within more components. Thus, based on the review of the literature which focuses on ToM as an understanding of multiple concepts rather than a single task paradigm (e.g., Pons et al., 2004; Wellman and Liu, 2004; Blijd-Hoogewys et al., 2008), we selected components that did not overlap and that represented different levels of difficulty. Children were therefore assessed for their perspective taking (two items in Level 1 and one item in Level 2), understanding of false-belief (three items), understanding of second-order false-belief (three items), recognition of basic emotion (five items), understanding of the impact of situational variations on emotions (five items), and understanding of desire-based emotion (two items). This choice avoided the tests to become too long, but warranted the inclusion of both visible or non-reflective dimensions of the mind and more invisible or reflective dimensions of the mind (Pons et al., 2009). For each item, the examiner showed a drawing while reading a story regarding the depicted characters, and the child was asked to attribute either a cognitive or an emotional mental state to the main character of the story by pointing to one of two or four possible answers. A composite score ranging from 0 to 21 was calculated by summing the number of correct items.

### Statistical Analyses

SPSS Statistics 22.0 was used for all analyses in the current study. First, preliminary analyses assessed the performances on the ToM tasks by age and gender through analysis of variance. Subsequently, the first hypothesis was examined through a mixed between-within-subjects analysis of variance to assess the impact of age, gender, and condition (individual and dyadic) on the performance in the Spatial task. To test the second hypothesis, correlation analysis and regression analysis were performed to assess the impact of ToM in explaining the variation on children’s spatial performance in the dyadic condition, while accounting for age, gender, and individual spatial performance.

## Results

**Table 1** shows the performances at the ToM tasks and at the Spatial task (individual and dyadic conditions) by age and gender. An analysis of variance Age X Gender indicated a significant and large effect of age on ToM performances (*F*(1,62) = 10.91, *p* = 0.002, η^2^ = 0.15), but no significant effect of gender or interaction between gender and age. Regardless of gender, older children had higher ToM performances than younger children.

**Table 1:** Theory of Mind (ToM) by age group and gender and Spatial Performance by condition, age group and gender.

			Spatial performance		ToM
				
Age group	Gender	*n*	Individual condition *M (SD)*	Dyadic condition *M (SD)*	*M (SD)*
Younger	Boys	18	3.1 (1.0)	4.7 (2.2)	17.6 (1.5)
	Girls	18	2.5 (0.85)	4.2 (1.3)	17.2 (1.6)
Older	Boys	14	3.1 (1.0)	4.4 (2.7)	18.4 (1.6)
	Girls	16	2.8 (0.71)	6.8 (1.7)	18.9 (1.2)
Total		66	2.9 (0.96)	5.0 (2.2)	18.1 (1.6)


### First Hypothesis

An analysis of variance Age X Gender X Condition showed a moderate effect of age (*F*(1,62) = 4.72, *p* = 0.034, η^2^ = 0.07), a large effect of condition (*F*(1,62) = 65.29, *p* = 0.000, η^2^ = 0.51), and no effect of gender on children’s performances on the Spatial task. The older children had higher performances (*M* = 4.3; *SD* = 1.7) than younger children (*M* = 3.7, *SD* = 1.6), regardless of condition and gender. Moreover, children had higher performances in the dyadic condition (*M* = 5.05, *SD* = 2.23) than in the individual condition (*M* = 2.92, *SD* = 0.96), regardless of age and gender. There was also an interaction effect of moderate size between age and gender (*F*(1,62) = 7.90, *p* = 0.007, η^2^ = 0.011), indicating that older girls performed better (*M* = 4.8, *SD* = 2.3) than older boys (*M* = 3.79, *SD* = 2.5), regardless the condition. An interaction of moderate effect size between gender and condition (*F*(1,62) = 6.62, *p* = 0.012, η^2^ = 0.10) furthermore showed that girls were better than boys in the dyadic setting, whereas there were no significant gender differences in the individual condition. Finally, a moderate interaction effect was found between condition, age, and gender (*F*(1,62) = 6.09, *p* = 0.016, η^2^ = 0.09), suggesting that older girls obtained higher scores than younger girls and they were better than boys from both age groups in the dyadic version of the Spatial task, but not in the individual condition.

### Second Hypothesis

Correlation analysis showed that ToM performances correlated with the spatial performances both in the individual (*r* = 0.26, *n* = 66, *p* < 0.038) and in the dyadic (*r* = 0.39, *n* = 66, *p* < 0.001) conditions, even when we control for age and gender (*r* = 0.26, *n* = 66, *p* < 0.038 and *r* = 0.32, *n* = 66, *p* < 0.010 for the individual and dyadic conditions, respectively). In the regression analysis, the role of age, gender, spatial performance in the individual condition, and scores in the ToM tasks for the performances on the Spatial task in the dyadic condition were examined. This regression model (Multiple *R* = 0.45, *F*(4,61) = 3.81, *p* < 0.008) showed that the predictors explained in total 20% (*R^2^* = 0.20) of the variation in the dependent variable. When examining the impact of the different predictors, ToM (*b* = 0.31; *t* = 2.4, *p* < 0.020), but not age, gender, nor spatial performance in the individual condition, had a significant effect on the spatial performance in the dyadic condition. ToM accounted for 15% of the shared variance (*r* = 0.39) and explained alone 8% (*r* = 0.29) of the variance of the children’s performance in the dyadic Spatial task.

## Discussion

The goal of this study was to investigate: (1) whether children improve their performance when resolving a Spatial task with a peer; and (2) whether individual differences in ToM affect children’s spatial performances in a dyadic setting. In line with prior research (Doise and Mugny, 1984; Psaltis and Duveen, 2007), we found that children improved their performance on the Spatial task when they resolved it together with a partner compared to when resolving it alone. For the first time, this study showed that children’s performances in a dyadic Spatial task were predicted by their ToM, even when accounting for age, gender, and the children’s spatial performances on the same task in an individual condition.

### Spatial Performances Across Age, Gender, and Condition

Confirming our first hypothesis, children performed better in the dyadic compared to the individual setting. This is consistent with the original experiments carried out by Doise and Mugny (1984) and other studies showing that children between 5 and 9 years of age profit from resolving tasks with a partner (e.g., Psaltis and Duveen, 2007; Zapiti and Psaltis, 2012). It has been argued that such results demonstrate that inter-individual conflicts are central for children’s cognitive development, and that this is particularly happening when children work on complementary resources to resolve problems (Buchs and Butera, 2004). The current study extends prior results on spatial problems that have reported beneficial effects of social interaction on cognitive performances in samples of older children and adults (Teasley, 1995; Tversky and Hard, 2009). One potential explanation is that the non-verbal and verbal behaviors of the other support the understanding of the objects and their spatial relations, so that the mutual action context promoted by social interaction helps children to (re-)think about the activity from the other’s perspective (Tversky and Hard, 2009; Frick and Wang, 2013).

As has been suggested earlier in the field (Piaget and Inhelder, 1952), the effect of age on children’s overall spatial performance indicates that spatial ability follows a developmental trend. The absence of an effect of age on spatial performance when children resolved the task by themselves might be related to the way we divided the groups. According to the literature, it is typically somewhere between the ages of 7 (younger group) and 9 (older group) years that children start to imagine an orientation outside their body, and work with relations such as before/behind and left/right (Piaget and Inhelder, 1952; Yilmaz, 2009). The enhanced performance of older compared to the younger children in the dyadic condition could be related to the higher reliance on more advanced social and linguistic abilities in this setting (Siegal, 2008).

The fact that gender had no main impact on children’s overall spatial performance contrasts with previous work that found that males perform better than females on mental rotation problems (Voyer et al., 1995; Yilmaz, 2009). However, this gender difference in mental rotation seems to appear from the age of 10, and could possibly be related to boys having more experiences with manipulation of symbolic information than girls by that age. Thus, gender differences may occur as the children gets older, which might explain the interaction effect between age and gender showing that older girls were better than younger boys, independent of the condition. Indeed, the literature suggests that the impact of gender varies according to both age and the type of task (Yilmaz, 2009), which shed some light on the interaction effect between gender and condition, and between age, gender, and condition. Thus, one reason for the gender differences in the dyadic setting may be that this condition depends more on broader social and language skills, which are dimensions where girls and older children typically demonstrate better abilities than boys and younger children (Walker et al., 2002; Siegal, 2008). More research with a larger age range is needed, however, to understand why gender differences appear in different conditions and how they might evolve over time.

### The Impact of ToM on Spatial Performances

The impact of age on ToM performances was expected, as previous studies have shown that ToM follows a clear developmental trend, both in boys and girls (e.g., Harris et al., 2005; Shahaeian et al., 2011). The results originally showed relations between ToM and spatial performances, both in the individual and in the dyadic conditions, even when age and gender were taken into account. Moreover, confirming the second hypothesis of this study, ToM had a positive impact on the spatial performance when children worked together, even when we controlled for age, gender, and spatial performance in the individual condition.

The link between ToM and the spatial performance in the individual setting indicates that the abilities to conceptually understanding the mind in terms of thoughts and emotions and to cognitively visualize objects in different positions based on an external frame of reference are related competences. The findings therefore expand previous results by demonstrating that understanding mental states has positive consequences not only on social competences (e.g., Roazzi et al., 2013; Farina and Belacchi, 2014) and the use of mental terms and metacognition (Veneziano et al., 2008; Lecce et al., 2010; Guajardo and Cartwright, 2016), but also on the domain of children’s cognition with regard to spatial visualization, which is a spatial transformation where “the positions of objects are moved with respect to an environmental frame of reference” (Hegarty and Waller, 2004, p. 127). In the present study it means that children with higher level of conceptual ToM were better able to mentally rotate the object and correctly transform the positions of the houses by taking the lake as the referential mark.

One could argue that once a relation between ToM and the spatial performance in the individual condition was found, a relation between ToM and the performance in the dyadic condition would be expected. Yet, the performance in the two conditions rely on different levels of spatial skills, as indicated by the findings showing the absence of a relation between the performance in the individual condition (making object-based transformation) and the performance in the dyadic condition (coordinating different perspectives). This is in line with the dissociation between tests of perspective taking and tests of mental rotation reported by others (Hegarty and Waller, 2004). Thus, we could not interpret the correlation between ToM and the performance in the dyadic condition as parallel to the correlation between ToM and the performance in the individual condition. It is also noteworthy that the relation between ToM and spatial performance was stronger in the dyadic compared with the individual setting. Moreover, beyond examining how ToM and the spatial performance in the dyadic condition were related, our aim was to investigate the degree to which ToM abilities could explain variation in the spatial performances in a social interaction setting. It was only ToM that significantly explained the performance in the Spatial task when children worked together, while the children’s age or their previous experience with the task did not. This finding therefore suggests the existence of socio-cognitive mechanisms underpinning spatial performance in social interactions.

A comparison of the two conditions of the Spatial task might deepen our understanding on such socio-cognitive mechanism. When resolving the task alone children had to visualize the houses in different positions by taking the lake as a reference. Even when the child changed the position to see the cardboard from a different angle (intra-individual conflict), the task in the individual setting centered around object-based transformations, while in the dyadic setting they needed to go beyond their own spatial visualization and deal with the other’s spatial perspective. In fact, the performance in the dyadic condition of the Spatial task seems to be more strongly dependent on the performance on the ToM tests where the child also had to take the mental perspective of the character. Thus, one could argue that a link between ToM and the spatial performance in the dyadic setting would be expected because the Spatial task in the dyadic condition essentially demands perspective taking. Nevertheless, the task in the dyadic condition cannot be reduced to its perspective taking dimension as the children also needed to manage the object-based transformation while coordinating different viewpoints with the other child, which is an advanced form of cognitive problem. In addition, we used a broad measure of ToM that assessed not only perspective taking but also false-belief and emotion comprehension, in which – different from the Spatial task – children’s beliefs and perspectives were not confronted by the experimenter or another child. Thus, the main explanation is that the findings add a new factor to the previous results on the reconstruction of the village task (e.g., Doise and Mugny, 1984; Zapiti and Psaltis, 2012) by pointing out that the better the child is at conceptually theorizing about the mind in a fictional scenario in terms of beliefs, perspectives, and emotions, the better he mentally rotates the objects while taking the spatial perspective of a real partner.

The current findings can therefore shed new light on the link between conceptual understanding of the mind and its practical implication for children’s cognition, especially for cognitive performance in social interaction. According to Tversky and Hard (2009), seeing another person in a scene near objects can elicit spontaneous perspective taking, which, in turn, create mutual expectations between partners while attempting to coordinate actions, imposed each person to go into multiple levels of perspectives. Nevertheless, Keysar et al. (2000) showed that even adults with high levels of ToM can demonstrate difficulties in applying these abilities to take other’s perspective. Accordingly, Samson and Apperly (2010) argue that using ToM could be a cognitively costly process involving the need to resist the interference from the egocentric perspective and to select relevant information necessary for ToM inferences, potentially creating a gap between competence and performance. We should point out some distinctions between the previous and the current findings. Notwithstanding the differences in age ranges, the aforementioned studies focused on perspective taking, while we have assessed a broad measure of ToM. This might suggest that the implication of ToM for children’s spatial performances cannot be seen as a uniform fact, as it can vary depending on the age range of the participants, how ToM is measured and what context it is applied in. A broad measure of ToM is potentially accounting for more variability in spatial performances than measures of perspective taking or false-belief alone, especially when the task is spatial and social at the same time (i.e., the village task). Perhaps a broad measure of ToM that includes the understanding of beliefs, desires, and emotions is part of a broader socio-cognitive process underlying spatial and social-perspective taking. In light of findings suggesting that social abilities are related to a more visually driven form of perspective taking (Clements-Stephens et al., 2013; Hamilton et al., 2014), future studies analyzing how children consider the other’s point of view while cooperatively resolving a spatial problem may contribute to understanding the extent to which and how ToM, social perspective taking and spatial performance are intertwined.

In sum, our results showed that conceptual competence can account for variation in cognitive performances on a Spatial task in children between 5–9 years of age, and in particularly so when the ToM measure includes different concepts. This does not indicate that we can directly translate ToM competence into spatial performance, and future studies should examine the role of potential third variables, such as language, cooperative behavior, intelligence, and executive functions (Wellman, 2014) to have a more complete picture of the role of ToM on spatial performance. As for now, the findings illustrate that, although not sufficient (Astington, 2003; Samson and Apperly, 2010), higher ToM levels can have positive implications for cognitive performances in terms of mental rotation and spatial perspective taking during peer interaction.

### Limitations

Some limitations should be mentioned. A larger sample size would have provided more power to detect significant relations and group differences in the present study. The inclusion of a post-test section (Doise and Mugny, 1984) would inform on possible long-term effects of the dyadic experiences. Future studies could also apply a longitudinal approach to address potential developmental processes. In addition, training studies aiming at strengthening ToM competences might provide stronger evidence of the positive impact of ToM on spatial performances. Inclusion of additional ToM concepts, as well as examination of the contributions of the separate components of the TEC and TMT could also contribute to a deeper understanding of the role of ToM on cognition.

Another limitation is that we did not analyze the interactional processes in the dyadic setting. Zapiti and Psaltis (2012), for instance, showed that what happens in the interaction affects the final spatial performance. In addition, Caputi et al. (2012) underlined that the relation between having and using ToM in social interaction is mediated by social factors. It could be argued that having the same intention toward the task does not specify the kind of social relation children would establish (Thomsen and Carey, 2013) and that different dyadic profiles, either more unilateral/hierarchical or more cooperative could affect performances in dyadic settings (Psaltis and Duveen, 2007). Thus, investigating the process of how children interact and operate with the socio-cognitive conflict could help to better understand how ToM explains the spatial performance in the dyadic Spatial task. Last, but not least, it is not certain that the same results would have occurred in other type of cognitive problem or if the spatial abilities were examined in a non-structured task. Investigating the impact of ToM in everyday interaction could deepen our understanding on the implication of ToM for children’s cognition with regard to the nature of the task and the nature of the interaction.

## Conclusion

Both hypotheses of the current study were confirmed: (1) children performed better in the dyadic setting compared to when doing it by themselves; and (2) children’s ToM had a positive impact on the spatial performance in the dyadic condition. Theoretically, these findings add a new aspect to the explanations based on inter-individual conflict and action-based reasoning (Doise and Mugny, 1984; Tversky and Hard, 2009; Zapiti and Psaltis, 2012) by illuminating socio-cognitive mechanisms that link conceptual competence in understanding the mind with spatial performance within interactional settings. The results demonstrate that individual differences in ToM – not only in terms of false-belief or perspective taking, but also in terms of emotion comprehension – impact children’s cognition and have to be taken into account in order to get a more complete picture of what promotes spatial performances in social interactions. Hence, three practical implications can be derived from it. First, it implies the need to elaborate more adequate and sensitive measures to grasp the cognitive consequences of ToM in a wide range of interactional contexts. Second, pedagogues might need to consider children’s ToM abilities when composing dyads and groups to solve spatial problems in cooperation, as such grouping might yield different outcomes. Finally, the findings suggest that teaching and strengthening of children’s ToM competences can have positive impact on children’s cognitive performance in important settings, such as in school, at least when it comes to spatial problems. To conclude, the link between what *ToM is* and what *ToM is for* (Liszkowski, 2013) does not indicate that ToM concepts are sufficient to efficiently promote successful cognitive outcome in social interaction (Astington, 2003). However, it shows that having such concepts goes beyond conceptual knowledge and can have practical implications for children’s cognition. This study demonstrates how this is the case in the domain of spatial transformation in peer interaction.

## Author Contributions

KV and FP designed the study. KV coordinated data collection and KV, IZ, EK, and FP contributed to the analysis and interpretation of the data for the work. KV prepared the first draft of the article and all authors revised it critically and approved the version to be published.

## Conflict of Interest Statement

The authors declare that the research was conducted in the absence of any commercial or financial relationships that could be construed as a potential conflict of interest.
